# Gastrointestinal Bleeding Induced by Immunoglobulin A Vasculitis on Post-Mild COVID-19 Patients

**DOI:** 10.7759/cureus.31212

**Published:** 2022-11-07

**Authors:** Daniel A Guifarro, Roberto Giron, Kathya Jimenez, Carlos Jose C Solis Almendarez, Eleazar E Montalvan Sanchez

**Affiliations:** 1 Facultad de Ciencias Médicas, Universidad Nacional Autónoma de Honduras, Tegucigalpa, HND; 2 Facultad de Medicina, Universidad Evangélica de El Salvador, San Salvador, SLV; 3 Internal Medicine, Hospital Escuela Universitario, Tegucigalpa, HND; 4 Department of Medicine, Indiana University School of Medicine, Indianapolis, USA

**Keywords:** steroids, iga vasculitis, covid-19, systemic vasculitis, gastrointestinal bleeding

## Abstract

We describe the case of a 51-year-old man who presented with a palpable purpuric rash and associated four days of lower gastrointestinal bleeding one month after testing positive for COVID-19. Urine studies showed evidence of microscopic hematuria and an increased protein/creatinine ratio.

An abdominal computed tomography scan showed distal ileitis, and a skin biopsy was significant for IgA vasculitis. Treatment with methylprednisolone was started, which led to the resolution of symptoms. Immunologic consequences of COVID-19 must not be overlooked, as they have a wide variety of presentations in diverse aged populations. IgA vasculitis is uncommon in adults, as well as gastrointestinal bleeding as a complication related to COVID-19.

## Introduction

As our experience with SARS-CoV-2 continues to grow, complications and immune-mediated consequences of the virus remain of interest. Several case reports have recently been published describing immunological alterations and autoimmune phenomena triggered by recent SARS-CoV-2 infection [1]. It is imperative to report these complications to increase prevention, awareness, and timely intervention. We describe a case of a patient that presented with atypical gastrointestinal bleeding after recently recovering from a SARS-CoV-2 infection. This case highlights the potential immunological complications seen with SARS-CoV-2 and the risk of acquired vasculitis.

## Case presentation

**Figure 1:**
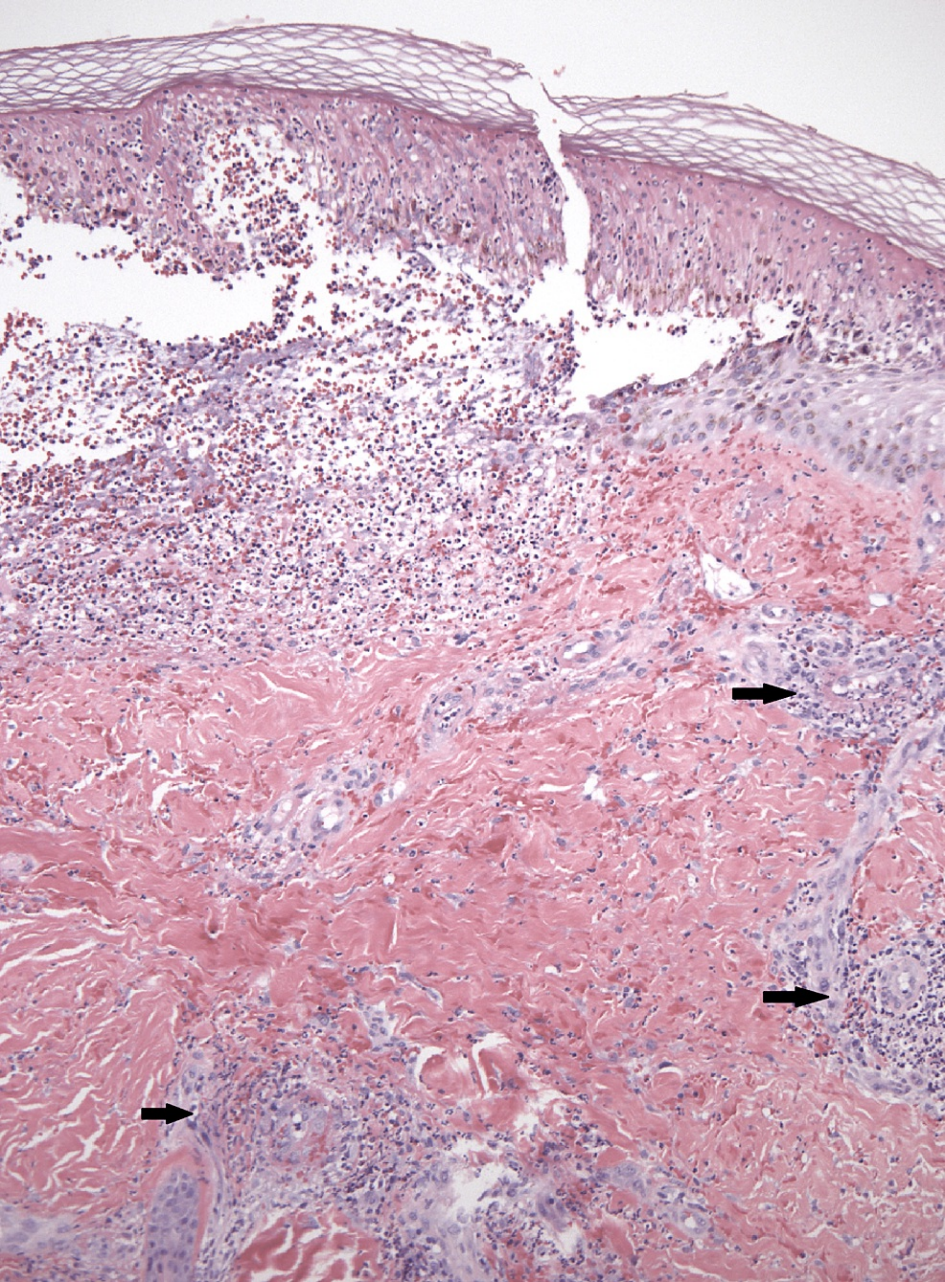
Skin biopsy. Skin biopsy showing surface neutrophil-rich pustule with leukocytoclastic vasculitis (black arrows).

A 51-year-old male with no medical and no COVID-19 vaccination history presented to the emergency department with a new-onset bilateral palpable purpuric rash on his lower extremities (Figure 1).

**Figure 2:**
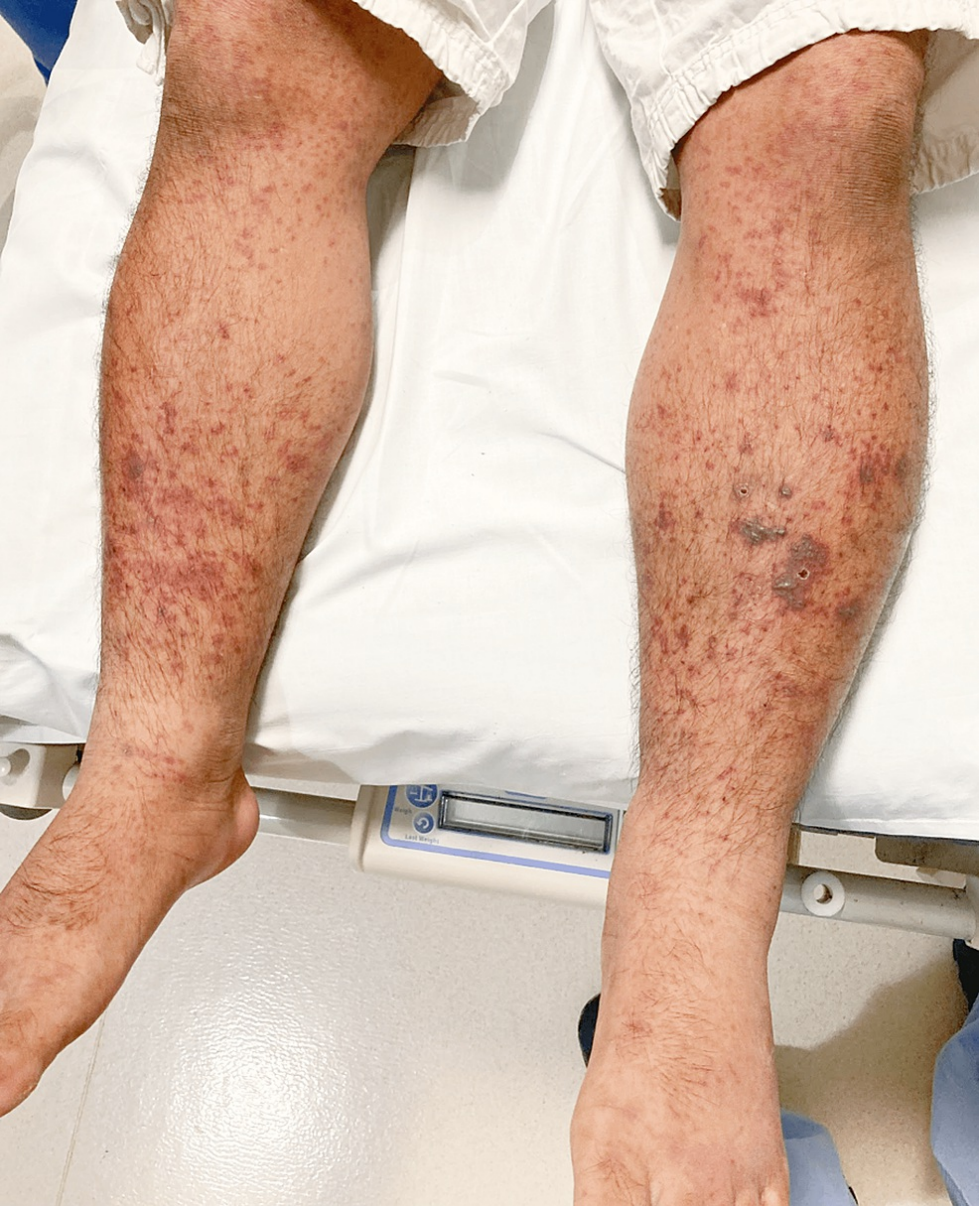
Painful palpable purpuric rash in lower extremities.

**Figure 3:**
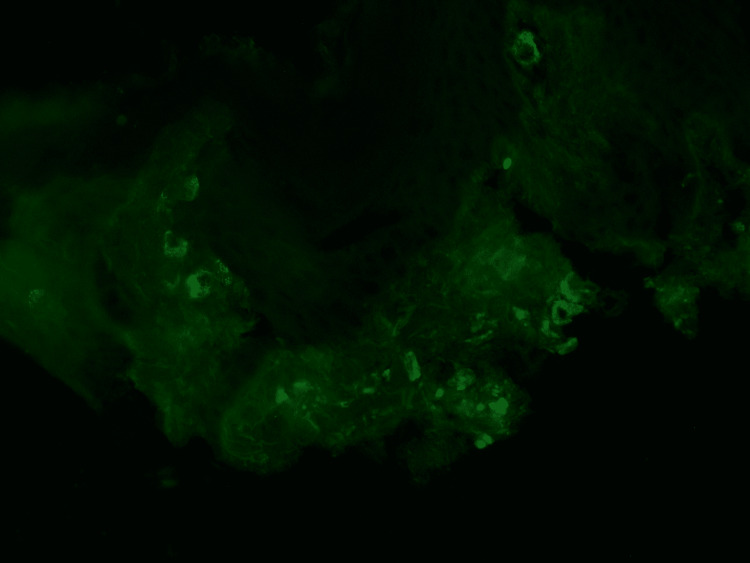
Direct immunofluorescence. Direct immunofluorescence showing granular perivascular IgA deposition.

Prior to hospitalization, the patient experienced four days of bright red blood in his stools. He denied any history of fever, weight loss, cough, shortness of breath, or joint pain. He did endorse mild intermittent diffuse abdominal pain. Furthermore, there was a recent history of travel to Southeast Asia, where he tested positive for SARS-CoV-2 via nasopharyngeal PCR swab after presenting hyposmia one month before the current symptoms. On physical examination, a palpable purpuric rash was present on the bilateral lower extremities, and fine pinpoint erythematous spots on the bilateral upper extremities. Digital rectal examination was negative. Urine studies showed microscopic hematuria and an increased protein/creatinine ratio of 708.3 mg/g. Remarkable laboratory results are included in Table 1. A computed tomographic (CT) angiogram scan of the abdomen and pelvis was suggestive of distal ileitis. The patient underwent a punch biopsy of the skin lesions from the left leg. A pathology result revealed IgA vasculitis (Figures 2, 3).

**Table 1:** General laboratories results ^1^ESR: Erythrocyte Sedimentation Rate, ^2^INR: International Normalized Ratio, ^3^PT: Prothrombin Time, ^4^ANA: Antinuclear Antibodies.

Table 1. General laboratories results	
Laboratory test	Patient value
Hemoglobin	11.5 g/dl
Platelets	240,000 mcL
ESR^1^	61 mm/hr
Ferritin	480 μ/L
INR^2^	1.10
PT^3^	12.9 sec
White blood cells	7.8 [10^9 L]
Albumin	2.8 μg/ml
Creatinine	0.86 mg/dl
Sodium	139 mEq/L
Potassium	3.7 mEq/L
ANA^4^	1:160

Screening for infectious diseases such as tuberculosis, HIV, Hepatitis B and C, and stool cultures were negative. The rheumatology workup was negative for serine proteinase 3, C3, C4, ANCA, and cryoglobulins.

Treatment was started with intravenous methylprednisolone 50 mg, which rapidly improved his rash in the first 24 hours. He was discharged home with an oral steroid taper and outpatient rheumatology follow-up. Over the next several weeks, his symptoms gradually improved. His rash had entirely resolved at the four-week follow-up, and he had no further episodes of bloody bowel movements. The hematuria also resolved, and proteinuria decreased from 701mg/dl to 251 mg/dl. 

## Discussion

When we approach autoimmune diseases, it is always important to investigate a possible trigger, which can be due to medications, drugs, or other diseases [2]. Many reports mention that SARS-CoV-2 is related to many autoimmune complications [3]. However, determining SARS-CoV-2 as a cause of autoimmune enteritis and IgA vasculitis can be difficult as various other causes can also cause it. An allergic reaction to a drug or a foreign agent is more likely. After other common causes of IgA vasculitis in a patient with a history of COVID-19 infection have been ruled out, SARS-CoV-2 should be considered. Studies already describe the relationship between SARS-CoV-2 and autoimmune complications, for example, rheumatological symptoms [4-5]. 

IgA vasculitis is a systemic, immune complex-mediated that generally develops after an upper respiratory infection and is accompanied by an abnormal IgA-mediated immune response [6]. IgA vasculitis is uncommon in the adult population, with an estimated incidence of 0.8-1.8/ 100,000 annually [7]. To our knowledge, few cases have been reported globally and have described the development of IgA vasculitis post-vaccination with COVID-19 and without vaccination. In the post-vaccination cases, gross hematuria was the most common manifestation, and skin lesions were relatively infrequent [8]. Jedlowski et al. describe five adult cases of post-COVID-19 IgA vasculitis unrelated to vaccination, in which adult males were predominantly affected, including symptoms involving the skin, joints, gastrointestinal tract, and renal systems [1]. Our case depicts a patient with a history of COVID-19 infection without previous vaccination, showing symptoms such as purpuric rash in the lower extremities and abdominal pain. He presented with GI bleeding, microscopic hematuria, and pathological findings of leukocytoclastic vasculitis.

Awareness of post-infectious autoimmune phenomena can help physicians provide high-quality healthcare as a prompt intervention in IgA vasculitis is vital to prevent end-stage renal disease [9]. In our patient, improvement of symptoms was achieved with early high-dose steroids, with complete resolution after four weeks, significantly decreasing this patient's risk of developing end-stage renal disease. Steroids may induce complete remission and prevent increased creatinine and lower protein excretion, with uncertain effects on preventing infections, malignancies, or death [10].

## Conclusions

This case report highlights the importance of considering SARS-CoV-2 as a possible cause of immune-mediated consequences. SARS-CoV-2 is still being studied, which means we are still discovering its relationship with other pathologies. The possibility for future studies that conclude a direct correlation between a patient with a recent history of SARS-CoV-2 and IgA vasculitis is an important fact to address in the future.
